# Draft Genome Sequences of Two Unclassified Bacteria, *Sphingomonas* sp. Strains IBVSS1 and IBVSS2, Isolated from Environmental Samples

**DOI:** 10.1128/genomeA.00894-17

**Published:** 2017-08-24

**Authors:** Russell J. S. Orr, Stephane Rombauts, Yves Van de Peer, Kamran Shalchian-Tabrizi

**Affiliations:** a Section for Genetics and Evolutionary Biology (EVOGENE), Department of Biosciences, University of Oslo, Oslo, Norway; b Department of Plant Biotechnology and Bioinformatics, Ghent University, Ghent, Belgium; c VIB Center for Plant Systems Biology, Ghent, Belgium

## Abstract

We report here the draft genome sequences of *Sphingomonas* sp. IBVSS1 and IBVSS2, two bacteria assembled from the metagenomes of surface samples from freshwater lakes. The genomes are >99% complete and may represent new species within the *Sphingomonas* genus, indicating a larger diversity than currently identified.

## GENOME ANNOUNCEMENT

Advances in high-throughput sequencing, coupled with decreasing costs, have led to the number of available bacterial genomes increasing almost exponentially. Genome sequencing, however, has traditionally been limited to species that can be held and grown in culture due to the high DNA volumes needed. A predominant focus on cultivable species has led to a genome bias, and true bacterial diversity is poorly represented. Metagenomic studies are rectifying this bias and have already revealed a large novel diversity (1). However, metagenomic studies remain limited, with many ecosystems yet to be sampled. We attempt to expand species richness in a bioproject with a goal to identify novel bacteria from various environmental samples. Here, we present the draft genomes of two unclassified *Sphingomonas* bacteria, isolated from the surface of freshwater lakes in Norway (Årungen, Ås) and Japan (Tsukuba, Ibaraki).

DNA was isolated using a standard phenol-chloroform protocol with ethanol precipitation and subsequent cleaning using Zymo genomic clean and concentrator. DNA was prepared and sequenced on the Illumina HiSeq2500 platform (150-bp paired-end reads; 350-bp insert size) and PacBio RS2 P6-C4 chemistry (20 kb) at the Norwegian Sequencing Centre. Metagenome drafts were assembled using SPAdes version 3.9.0 (2), with single genomes separated with MetaBAT (3), and quality was assessed with CheckM (4). Separate genomes were scaffolded using LINKS (5), and gaps were closed with Sealer (6). Genome assemblies were evaluated with PROmer (7) and REAPER (8) before being improved with Pilon (9). Genomes were annotated using the NCBI Prokaryotic Genome Annotation Pipeline (10). Taxonomical rank was established on evaluation of CheckM (4), PhyloSift (11), and megaBLAST against the NCBInr database.

*Sphingomonas* sp. IBVSS1 was assembled into 11 scaffolds, constituting 14 contigs with a sequence length of 3.15 Mb and a GC content of 66.67%. The scaffold *N*_50_ value was 0.73 Mb with Illumina coverage of 12× and PacBio coverage of 13×. CheckM estimated genome completeness at 99.54% with no contamination or strain heterogeneity. The genome constitutes 2,909 genes, 49 RNAs, 43 tRNAs, 3 noncoding RNAs (ncRNAs), and 50 pseudogenes.

*Sphingomonas* sp. IBVSS2 was assembled into nine scaffolds, constituting 10 contigs with a total sequence length of 4.29 Mb and a GC content of 67.81%. The scaffold *N*_50_ value was 0.75 Mb with Illumina coverage of 84× and PacBio coverage of 16×. CheckM estimated genome completeness at 99.54% with no contamination or strain heterogeneity. The genome constitutes 3,925 genes, 52 RNAs, 46 tRNAs, 3 ncRNAs, and 29 pseudogenes.

Genomes were confirmed as novel *Sphingomonas* spp. by using 16S queries to perform a BLASTn search against the NCBInr database: IBVSS1 had a 95% identity to *S. sanxanigenens* (GenBank accession number CP006644), and IBVSS2 had a 96% identity to the 16S rRNA of *S. taxi* (CP009571). IBVSS1 and IBVSS2 had a 92% 16S rRNA identity to each other. The low identity to known *Sphingomonas* bacteria may suggest IBVSS1 and IBVSS2 as new species, indicating a larger diversity than currently identified.

### Accession number(s).

The draft genomes of *Sphingomonas* sp. strains IBVSS1 and IBVSS2 sequenced under this project have been deposited at DDBJ/EMBL/GenBank under the accession numbers NFUS00000000 and NFUR00000000, respectively. These biosamples (SAMN06840509 and SAMN06840510, respectively) are part of BioProject PRJNA384425.

## References

[1] Nayfach S, Pollard KS 2016 Toward accurate and quantitative comparative metagenomics. Cell 166:1103–1116. doi:10.1016/j.cell.2016.08.007. 27565341 PMC5080976

[2] Bankevich A, Nurk S, Antipov D, Gurevich AA, Dvorkin M, Kulikov AS, Lesin VM, Nikolenko SI, Pham S, Prjibelski AD, Pyshkin AV, Sirotkin AV, Vyahhi N, Tesler G, Alekseyev MA, Pevzner PA 2012 SPAdes: a new genome assembly algorithm and its applications to single-cell sequencing. J Comput Biol 19:455–477. doi:10.1089/cmb.2012.0021. 22506599 PMC3342519

[3] Kang DD, Froula J, Egan R, Wang Z 2015 MetaBAT, an efficient tool for accurately reconstructing single genomes from complex microbial communities. PeerJ 3:e1165. doi:10.7717/peerj.1165. 26336640 PMC4556158

[4] Parks DH, Imelfort M, Skennerton CT, Hugenholtz P, Tyson GW 2015 CheckM: assessing the quality of microbial genomes recovered from isolates, single cells, and metagenomes. Genome Res 25:1043–1055. doi:10.1101/gr.186072.114. 25977477 PMC4484387

[5] Warren RL, Vandervalk BP, Jones SJ, Birol I 2015 LINKS: scaffolding genome assemblies with kilobase-long nanopore reads. bioRxiv doi:10.1101/016519.

[6] Paulino D, Warren RL, Vandervalk BP, Raymond A, Jackman SD, Birol I 2015 Sealer: a scalable gap-closing application for finishing draft genomes. BMC Bioinformatics 16:230. doi:10.1186/s12859-015-0663-4. 26209068 PMC4515008

[7] Delcher AL, Phillippy A, Carlton J, Salzberg SL 2002 Fast algorithms for large-scale genome alignment and comparison. Nucleic Acids Res 30:2478–2483. doi:10.1093/nar/30.11.2478. 12034836 PMC117189

[8] Hunt M, Newbold C, Berriman M, Otto TD 2014 A comprehensive evaluation of assembly scaffolding tools. Genome Biol 15:R42. doi:10.1186/gb-2014-15-3-r42. 24581555 PMC4053845

[9] Walker BJ, Abeel T, Shea T, Priest M, Abouelliel A, Sakthikumar S, Cuomo CA, Zeng Q, Wortman J, Young SK, Earl AM 2014 Pilon: an integrated tool for comprehensive microbial variant detection and genome assembly improvement. PLoS One 9:e112963. doi:10.1371/journal.pone.0112963. 25409509 PMC4237348

[10] Angiuoli SV, Gussman A, Klimke W, Cochrane G, Field D, Garrity GM, Kodira CD, Kyrpides N, Madupu R, Markowitz V, Tatusova T, Thomson N, White O 2008 Toward an online repository of standard operating procedures (SOPs) for (meta) genomic annotation. OMICS 12:137–141. doi:10.1089/omi.2008.0017. 18416670 PMC3196215

[11] Darling AE, Jospin G, Lowe E, Matsen FA, Bik HM, Eisen JA 2014 PhyloSift: phylogenetic analysis of genomes and metagenomes. PeerJ 2:e243. doi:10.7717/peerj.243. 24482762 PMC3897386

