# Erratum for Orr et al., “Draft Genome Sequences of Two Unclassified Bacteria, *Sphingomonas* sp. Strains IBVSS1 and IBVSS2, Isolated from Environmental Samples”

**DOI:** 10.1128/genomeA.01175-17

**Published:** 2017-10-12

**Authors:** Russell J. S. Orr, Stephane Rombauts, Yves Van de Peer, Kamran Shalchian-Tabrizi

**Affiliations:** a Section for Genetics and Evolutionary Biology (EVOGENE), Department of Biosciences, University of Oslo, Oslo, Norway; b Department of Plant Biotechnology and Bioinformatics, Ghent University, Ghent, Belgium; c VIB Center for Plant Systems Biology, Ghent, Belgium

## ERRATUM

Volume 5, no. 34, e00894-17, 2017, https://doi.org/10.1128/genomeA.00894-17. Page 2: Reference 5 should read as follows.

5. Warren RL, Yang C, Vandervalk BP, Behsaz B, Lagman A, Jones SJ, Birol I. 2015. LINKS: scalable, alignment-free scaffolding of draft genomes with long reads. Gigascience 4:35. https://doi.org/10.1186/s13742-015-0076-3.

